# Anti-H7N9 avian influenza A virus activity of interferon in pseudostratified human airway epithelium cell cultures

**DOI:** 10.1186/s12985-019-1146-4

**Published:** 2019-04-03

**Authors:** Ai-jun Chen, Jie Dong, Xin-hui Yuan, Hong Bo, Shu-zhen Li, Chao Wang, Zhao-jun Duan, Li-shu Zheng

**Affiliations:** 1 National Institute for Viral Disease Control and Prevention, China CDC, Key Laboratory for Medical Virology National Health Commission, 100 Ying-Xin St., Xi-Cheng District, Beijing, 100052 China; 2 The First Hospital of Lanzhou University, Lanzhou, 730000 China; 3 Nanjing Key Laboratory of Pediatrics, Children’s Hospital of Nanjing Medical University, Nanjing, 210008 China; 4 National Institute for Viral Disease Control and Prevention, China CDC, NHC Key Laboratory of Medical Virology and Viral Diseases, 100 Ying-Xin St., Xi-Cheng District, Beijing, 100052 China

**Keywords:** Avian influenza A virus, H7N9, Human airway epithelium, Interferon, Interferon-stimulated genes

## Abstract

**Background:**

Since H7N9 influenza A virus (H7N9) was first reported in 2013, five waves of outbreaks have occurred, posing a huge threat to human health. In preparation for a potential H7N9 epidemic, it is essential to evaluate the efficacy of anti-H7N9 drugs with an appropriate model.

**Methods:**

Well-differentiated pseudostratified human airway epithelium (HAE) cells were grown at the air–liquid interface, and the H7N9 cell tropism and cytopathic effect were detected by immunostaining and hematoxylin-eosin (HE) staining. The H7N9 replication kinetics and anti-H7N9 effect of recombinant human α2b (rhIFN-α2b) and rhIFN-λ1 were compared with different cell lines. The H7N9 viral load and interferon-stimulated gene (ISG) expression were quantified by real-time PCR assays.

**Results:**

H7N9 could infect both ciliated and non-ciliated cells within the three-dimensional (3D) HAE cell culture, which reduced the number of cilia and damaged the airways. The H7N9 replication kinetics differed between traditional cells and 3D HAE cells. Interferon had antiviral activity against H7N9 and alleviated epithelial cell lesions; the antiviral activity of rhIFN-α2b was slightly better than that of rhIFN-λ1. In normal cells, rhIFN-α2b induced a greater amount of ISG expression (MX1, OAS1, IFITM3, and ISG15) compared with rhIFN-λ1, but in 3D HAE cells, this trend was reversed.

**Conclusions:**

Both rhIFN-α2b and rhIFN-λ1 had antiviral activity against H7N9, and this protection was related to the induction of ISGs. The 3D cell culture model is suitable for evaluating interferon antiviral activity because it can demonstrate realistic in vivo-like effects.

## Background

In addition to the seasonal influenza virus, some avian influenza viruses, such as H7N9 and H5N1 avian influenza A, can also infect humans. Since the first human infection with a novel H7N9 influenza virus (H7N9) was confirmed in China in the spring of 2013 [1], there have been five epidemic waves of human H7N9 infections through 2017 [2]. Vaccination is the most effective way to prevent influenza infection. However, there is currently no commercial human vaccine available that is specific for H7N9. Antiviral treatment is another critical strategy for controlling infection with H7N9, and neuraminidase (NA) inhibitors are the most widely used drugs against influenza infection [3]. However, with the increase in drug-resistance-conferring mutations, other measures are also needed to treat infection with H7N9. Previous studies have shown that type I interferon (IFN) was active against the influenza 2009 pandemic H1N1 and highly pathogenic H5N1 strains [4, 5]. Additionally, the natural IFN Alferon N, was shown to inhibit the replication of oseltamivir-sensitive and -resistant H7N9 isolates [6].

Primary human airway epithelium (HAE) cells can be further differentiated into polarized HAE cells when they are subjected to air–liquid interface (ALI) culture for 4 to 6 weeks. The morphology and functionality of these cells resembles the in vivo human pseudostratified mucociliary epithelium, and this system is a promising tool for the study of respiratory viruses. Many common and emerging respiratory viruses, such as influenza A [7, 8], respiratory syncytial virus (RSV) [9], adenovirus (ADV) [10], parainfluenza virus (PIV) [11], and human coronavirus (HCoV) [8, 12], can replicate in these three-dimensional (3D) HAE cells. Moreover, some newly described viruses, including HBoV [13, 14] and HCoV HKU1 [15], that could not be cultured on traditional cell lines can be cultured on these cells. Of the currently available cell culture models, 3D HAE cells reconstruct the morphological and physiological characteristics of the respiratory tract to the greatest extent, therefore, it is a robust cellular model for respiratory virus research and can also be used to evaluate the therapeutic effect of drugs and transgenic strategies [16].

Despite type I and type III IFN binding to different receptors, they both use similar JAK–STAT signal pathways and induce the expression of an overlapping set of IFN-stimulated genes (ISGs) [17]. Thus, the type III IFNs shares some properties with the type I IFNs, such as a role in antiviral defense as well as antiproliferative and immunoregulative activities [18].

In this study, we used 3D HAE cell cultures to study the target cell tropism and the infection and proliferation features of H7N9. The antiviral activities of type III and type I recombinant human IFNs (rhIFNs) were compared on A549 cells, 2D HAE cells, and 3D HAE cells, and the expression of antiviral genes in different cell models was also analyzed.

## Materials and methods

### Virus and cells

H7N9 A/Anhui/1/2013 was obtained from the Chinese National Influenza Center, and all experiments with this virus were performed in approved enhanced biosafety level 3 (BSL-3) laboratories. A549 cells were cultured in Dulbecco’s modified Eagle medium (DMEM; Gibco, NY, USA) with 10% fetal bovine serum (Gibco).

### Human airway epithelial cell culture

Primary HAE cells were isolated from patients who underwent surgical lung resection for pulmonary diseases in Nanjing Children’s Hospital, as described previously [19]. HAE cells were plated onto type I and III collagen-coated six-well tissue culture plates and cultured in BEGM media (Lonza, Germany) supplemented with the required additives (Lonza). When the cells reached 80–90% confluence, traditional monolayer two-dimensional (2D) HAE cells were dissociated with trypsin, and 3 × 10^5^ cells were seeded on type IV collagen-coated 12-well transwell inserts (Costar, ME, USA). Medium was renewed for both the apical and basolateral surfaces every other day. The medium was then changed to air–liquid interface (ALI) medium (BEGM+DMEM+additives) until the HAE cells reached full confluence. After 5 days, the HAE cells were exposed to air, and only the basolateral compartment was cultured with ALI medium. The ALI culture was continued for 4–6 weeks, during which time the cells differentiated into 3D pseudostratified HAE cells. Prior to the experiments, all cultures were maintained at 37 °C in a 5% CO_2_ incubator.

### Immunostaining for H7N9 virus on 3D HAE cells

3D HAE cells that had been infected with 100 tissue culture infective doses (TCID_50_) of H7N9 for 24 h were fixed with 4% paraformaldehyde (PFA) for 30 min at room temperature, followed by washing of both the apical and basolateral sides three times each with PBS. Subsequently, the fixed cells were permeabilized with 0.2% Triton X for 2 h and blocked with 5% bovine serum albumin for 1 h. For the simultaneous detection of H7N9 protein M2 and ciliated cells or tight junctions, anti-influenza A M2 rabbit polyclonal antibody (PA5–32233, 1:500, Thermo Fisher), and mouse monoclonal anti-β tubulin antibody (T4026, 1:100, Sigma) or mouse monoclonal anti-ZO-1 antibody (33–9100, 1:500, Invitrogen, CA, USA), respectively, were applied as primary antibodies. Goat-derived Dylight 594-labeled anti-mouse IgG (H + L) (35,511, 1:500, Thermo Fisher) and Dylight 488-labeled anti-rabbit IgG (H + L) (35,553, 1:500, Thermo Fisher) were applied as secondary antibodies. Nuclei were counterstained with DAPI. Finally, the filters with cells were excised from the insert and mounted under coverslips on glass slides with mounting medium. Confocal images were taken with an UltraView VoX confocal microscope (PerkinElmer, Boston, MA, USA).

### Inoculation of H7N9 virus on different cell lines

The apical surface of 3D HAE cells was rinsed three times with PBS and then incubated with 100 μl of 0.25 μg/ml rhIFN-λ1 (R&D, USA) or 60 IU/ml rhIFN-α2b (Yuance, China) for 20 h at 37 °C in a 5% CO_2_ incubator. After discarding the cell culture supernatant, the 3D HAE cells were inoculated on the apical surface with 100 μl of 1 TCID_50_ of H7N9 per well and incubated for 1 h. Virus and rhIFNs diluents were prepared with ALI medium. At the end of the incubation, the unbound H7N9 viral particles were removed by rising with Hank’s buffer, and the basolateral compartment was supplemented with 1.5 ml of ALI medium for 24 h at 37 °C in a 5% CO_2_ incubator. Subsequently, 200 μl of ALI medium was added to the apical surface, and the supernatants were collected after 30 min. Virus RNA was extracted using the QIAamp MinElute Virus Spin Kit (Qiagen, Hilden, Germany), and quantification of H7N9 viral yields was performed using AgPath-ID™ One-Step RT-PCR Reagents (ABI, USA) to analyze the anti-H7N9 effect of different rhIFNs.

For A549 cells, the experimental method was similar to that used for 3D HAE cells, except that the A549 cells were cultured on 24-well plates and the applied virus medium was DMEM with added TPCK-trypsin (0.5 μg/ml). Nucleic acids were extracted and quantified once per day for 10 days. Real-time PCR assays were used for the detection of the HA gene from H7N9 A/Anhui/1/2013. The primer sequences were H7N9-F 5′-AGAAATGAAATGGCTCCTGTCAA-3′ and H7N9-R 5′-GGTTTTTTCTTGTATTTTTATATGACTTAG-3′, and the probe sequence was H7N9-P FAM-AGATAATGCATTCCCGCAGAT-TAMRA.

### Anti-H7N9 activity of different types of rhIFNs

2D HAE cells were rinsed three times with PBS and then incubated with 100 μl of successive 4^0^- to 4^9^-fold dilutions of 4 μg/ml rhIFN-λ1 (R&D) or 1000 U/ml rhIFN-α2b (Yuance) for 20 h at 37 °C in a 5% CO_2_ incubator. Next, the cells were challenged with 100 μl of 100 TCID_50_ of H7N9 per well. After incubation for 1 h, the unbound H7N9 viral particles were removed by rising with Hank’s buffer, and the cells were supplemented with 100 μl of BEGM medium. Harvests were collected at 72 h post-inoculation, and H7N9 RNA extraction and nucleic acid quantification were performed as described above to analyze the anti-H7N9 effect of different rhIFNs. For 3D HAE cells and A549 cells, the experimental protocols were the same as that described for 2D HAE cells, but the medium used for cell culture was ALI medium and DMEM, respectively.

### HE staining

The apical surface and the basolateral chamber of 3D HAE cells were cultured with 4 μg/ml rhIFN-λ1 (R&D, USA) or 1000 IU/ml rhIFN-α2b (Yuance, China) for 20 h at 37 °C in a 5% CO_2_ incubator (500 μl and 1.5 ml for the apical surface and basolateral chamber, respectively). The apical surface was washed three times with PBS, followed by inoculation with 100 μl of 100 TCID_50_ of H7N9, and the basolateral chamber was maintained with 1.5 ml of ALI medium. After incubation for 1 h, the apical chamber was washed twice with PBS and maintained continuously with an ALI for 24 h. The cells were fixed in 4% PFA along with the entire transwell insert for hematoxylin-eosin (HE) staining.

### Detection of ISGs

A549 cells, 2D HAE cells, and well-differentiated pseudostratified 3D HAE cells were each incubated with 60 IU/ml rhIFN-α2b (Yuance) or 0.25 μg/ml rhIFN-λ1 (R&D) for 18 h at 37 °C in a 5% CO_2_ incubator. After rinsing with PBS, the cells were lysed with Trizol, and the total RNA was extracted. Reverse transcription was performed using the Superscript® III First-Strand Synthesis System (Invitrogen). The relative expression levels of ISGs, including ISG15, MX1, and OAS1, were analyzed by a Taqman real-time PCR assay using the 2^-ΔΔCT^ method, and GADPH was used as the internal reference [20]. The sequences of primers and probes are shown in Table 1.

**Table 1:** The ISG primers and probes used for real-time PCR

Gene	Sequence of primers and probes
GADPH	F: GAAGGTGAAGGTCGGAGTC R: GAAGATGGTGATGGGATTTC P: FAM-AAGGTCGGAGTCAACGGATTTGGTC-TAMRA
ISG15	F: GGACAAATGCGACGAACCTC R: GCTCACTTGCTGCTTCAGG P: FAM-CCCGCCAGCATCTTCACCGT-TAMRA
MX1	F: TGCAGGACAAGGACACCTAC R: TCACCACGGCTAACGGATAA P: FAM-ATAAAGCCCAGAATGCCATCGCC-TAMRA
OAS1	F: AGCATTTTCCGTGAAGTTTG R: GGGTTAGGTTTATAGCCGCC P: FAM-AGAGGCCGATCTGACGCTGAC-TAMRA
IFITM3	F: GAGAACCATCCCAGTAACCC R: GACAGGAGAGAAGAAGGTTTGG P: FAM-TTCGCTGGACACCATGAATCACACT-TAMRA

## Results

### Immunofluorescence analysis

**Fig. 1:**
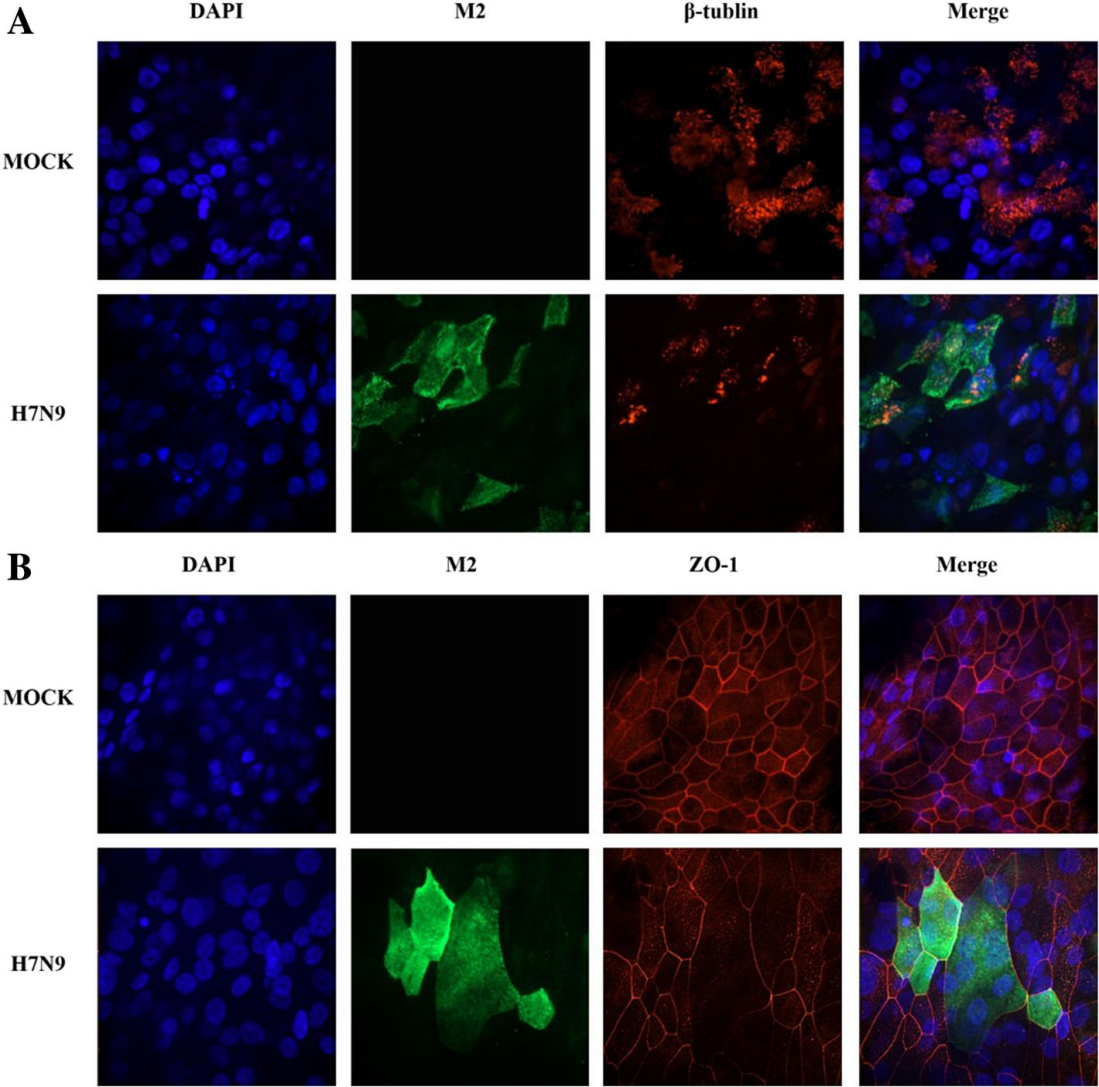
Immunofluorescence of 3D HAE cells infected with H7N9. At 24 h post-inoculation with H7N9, the 3D HAE cells were fixed, followed by double immunostaining with anti-influenza A M2 polyclonal antibody (green) and mouse monoclonal anti-β tubulin antibody (red, **a**) or mouse monoclonal anti-ZO-1 antibody (red, **b**). Confocal images were taken with a magnification of 100×. Nuclei were stained with DAPI

β-tubulin is an important component of the cytoskeleton and is a marker protein of cilia, so it can be used to distinguish between ciliated cells and non-ciliated cells. The tight junction protein zonula occludens-1 (ZO-1) is located at the apex of epithelial cells; it is a major component of tight junctions and plays an important role in maintaining pseudostratified cell layer integrity and barrier function. As shown by immunofluorescence staining with anti-β-tubulin IV (Fig. 1a) and anti-ZO1 (Fig. 1b) antibodies, H7N9 could infect both non-ciliated and ciliated cells. In contrast with the state of the cilia in the mock control, 3D HAE cells that were infected with H7N9 showed an obvious reduction of cilia. Additionally, the H7N9-infected 3D HAE cells showed a disassociation of the ZO-1, suggesting airway epithelial damage.

### Replication characteristics of H7N9 influenza virus on different cells

**Fig. 2:**
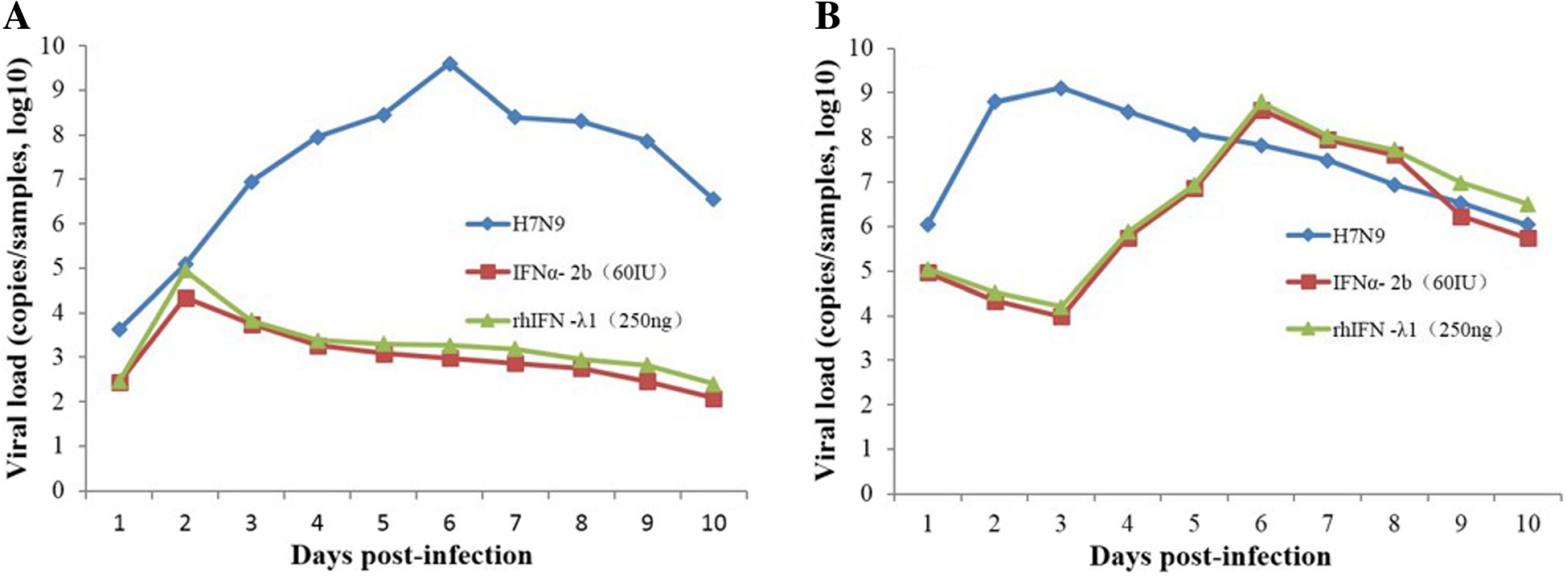
Replication characteristics of H7N9 on A549 cells and 3D HAE cells. A549 cells (**a**) and 3D HAE cells (**b**) were each incubated with 0.25 μg/ml rhIFN-λ1 or 60 IU/ml rhIFN-α2b for 20 h and then infected with 1 TCID_50_ of H7N9 virus. Viral RNA was extracted once per day for 10 days. The H7N9 HA gene was quantitatively detected by real-time PCR

A549 cells and 3D HAE cells were each challenged with 1 TCID_50_ of H7N9 after being treated with rhIFN-α2b (60 IU/ml) or rhIFN-λ1 (250 ng/ml) for 20 h. The viral load was then detected by real-time PCR for 10 consecutive days using the method described above. In the control group without rhIFN treatment, the viral load in A549 cells reached a peak of 3.9 × 10^9^ copies/ml on the sixth day and decreased to 3.5 × 10^6^ copies/ml on the tenth day (Fig. 2a). In control 3D HAE cells, the viral load reached a peak of 1.29 × 10^9^ copies/ml on the third day and was reduced to 1.1 × 10^6^ copies/ml on the tenth day (Fig. 2b). In contrast, the viral load of A549 cells treated with rhIFN peaked on the second day and then gradually decreased (Fig. 2a). For 3D HAE cells, viral replication was inhibited during the first few days after rhIFN treatment; the viral load was reduced to 1.58 × 10^4^ copies/ml (rhIFN-α2b) or 9.55 × 10^3^ copies/ml (rhIFN-λ1) on the third day. Later, it gradually rose to a peak of 4.26 × 10^8^ copies/ml (rhIFN-α2b) or 6.31 × 10^8^ copies/ml (rhIFN-λ1) on the sixth day and then gradually decreased again (Fig. 2b).

### Anti-H7N9 activity of rhIFNs on different cells

**Fig. 3:**
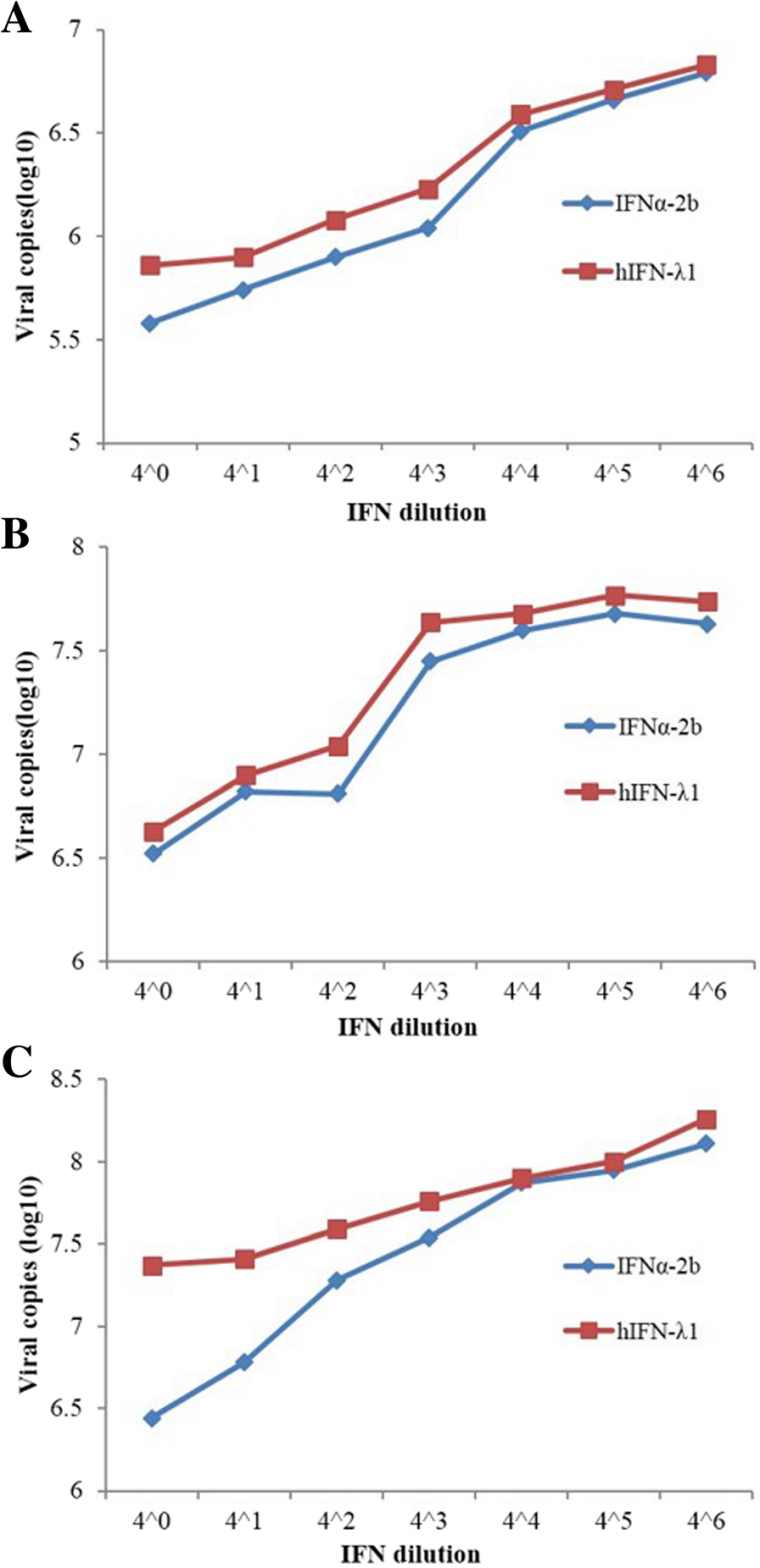
Anti-H7N9 bioactivity of different rhIFN types. A549 cells (**a**), 2D HAE cells (**b**), and 3D HAE cells (**c**) were each incubated with successive 4^0^- to 4^9^-fold dilutions of 4 μg/ml rhIFN-λ1 or 1000 IU/ml rhIFN-α2b for 20 h and then challenged with 100 TCID_50_ of H7N9. At 72 h post-inoculation, anti-H7N9 bioactivity of different rhIFNs were compared as described above

A549 cells, 2D HAE cells and 3D HAE cells were each incubated with serial dilutions of rhIFNs (initial concentration of rhIFN-λ1 and rhIFN-α2b was 4 μg/ml and 1000 IU/ml, respectively) for 20 h, after which these cells were challenged with 100 TCID_50_ of H7N9. Viral RNA was extracted 72 h later, and quantitative real-time PCR assay targeting the H7N9 HA gene was conducted to quantify the viral load. Among the three cell types, viral proliferation was the fastest in 3D HAE cells, reaching 1 × 10^10^ copies/ml, while the HA gene copy number in A549 cells and 2D HAE cells was 2.5 × 10^7^ copies/ml and 8 × 10^7^ copies/ml, respectively. The antiviral activity of rhIFN-α2b was superior to that of rhIFN-λ1 in all three cell types, A549 cells (Fig. 3a), 2D HAE cells (Fig. 3b), and 3D HAE cells (Fig. 3c). As the dose of rhIFN decreased, the antiviral activity also decreased, which demonstrates a dose-response relationship. Compared with the control group, the viral load was decreased to 2.8 × 10^6^ copies/ml and 2.3 × 10^7^ copies/ml, which means 3571-fold and 435 -fold (1 × 10^10^ copies/ml) reduction, respectively, when the 3D HAE cells were treated with 4 μg/ml rhIFN-λ1 or 1000 IU/ml rhIFN-α2b. Because of the biggest drop in viral load, the antiviral activity of rhIFN was the strongest when it was applied to the 3D cells (Fig. 3c).

### Cytopathic effect of H7N9 on 3D HAE cells

**Fig. 4:**
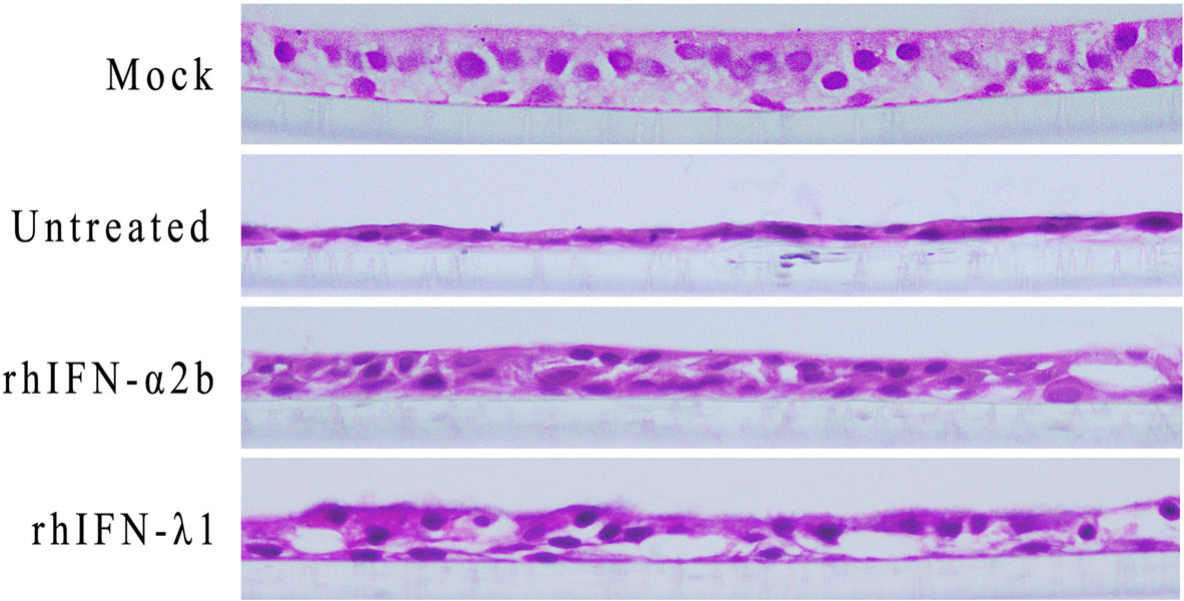
Cytopathic effect of rhIFN on 3D HAE cells. After being incubated for 24 h with rhIFN-λ1 or rhIFN-α2b, the 3D HAE cells were infected with 100 TCID_50_ of H7N9 for 24 h. The cells were then fixed and stained with HE, and images were taken with a microscope at a magnification of 40×

HE staining of ALI-cultured HAE cells revealed that the nuclear position was uneven, there appeared to be multi-layered cells with cilia distributed at the top, and the morphological structure was similar to the pseudostratified ciliated columnar epithelium of human tracheal tissue. In the H7N9 infection group, there were many vacuoles, the number of cilia was reduced, and the cell layer was thinner. However, when 3D HAE cells were incubated with rhIFNs and then challenged with H7N9, the cytopathic effect of H7N9 infection was alleviated (Fig. 4).

### rhIFNs-induced gene expression

**Fig. 5:**
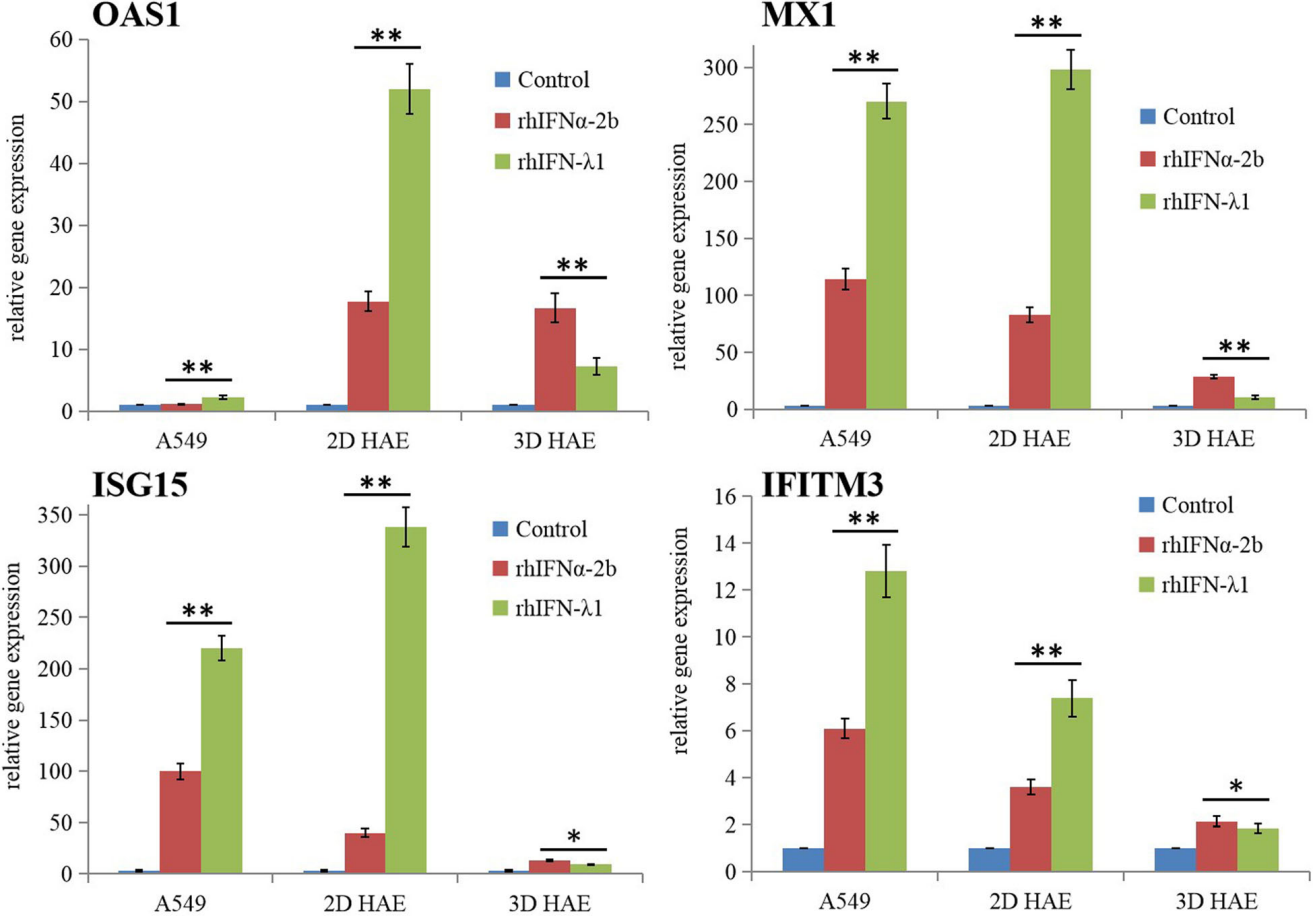
Effect of rhIFN treatment on ISG induction in different cells. After treatment of A549 cells, 2D HAE cells, and 3D HAE cells with rhIFN-α2b or rhIFN-λ1, the total RNA was extracted and reverse transcribed. The relative expression levels of ISG15, MX1, OAS1, and IFITM3 were measured using Taqman real-time PCR assays and quantified by the 2^−ΔΔCT^ method. *P < 0.05; **P < 0.01

The relative expression levels of OAS1, MX1, ISG15, and IFITM3 were detected by real-time PCR using GADPH as the internal reference. Compared with the control group without rhIFNs treatment, cells treated with either rhIFN-α2b or rhIFN-λ1 had significantly upregulated expression levels of all four genes. For A549 cells and 2D HAE cells, the expression levels of these four genes were significantly higher in the rhIFN-λ1 group than in the rhIFN-α2b group. In contrast, the ISG expression levels were significantly higher in the rhIFN-α2b group than in the rhIFN-λ1 group for the 3D HAE cells (Fig. 5). These results demonstrate that the gene expression induced by rhIFNs was different between 3D HAE cells and ordinary 2D-cultured cells.

## Discussion

The human population has little or no immunity to H7N9 because the H7 subtype of influenza has never previously infected humans; thus, infection with H7N9 may have a high mortality rate [21]. Since 2013, H7N9 has caused five outbreak waves, and 1561 laboratory-confirmed cases of H7N9 infection have been reported [22]. H7N9 has also been detected in animals and the environment; therefore, it poses a significant threat to public health as well as a potential pandemic risk. Currently, no universal influenza vaccine is available, and NA inhibitors are the only FDA-approved drugs for the treatment of H7N9 infection [23]. With the rapid emergence of NA inhibitor-resistant strains after treatment with oseltamivir, the utility of NA inhibitors in the clinical treatment of influenza-infected patients is limited, so other drugs will be required to control H7N9 infection.

In 1988, a tracheal epithelial cell model with a polarity of differentiation was established via ALI culture [24]. Subsequently, this model has been widely used for the amplification, isolation, and identification of respiratory viruses, as well as for the study of respiratory viral infection and therapeutic mechanisms. Most importantly, the 3D HAE system greatly facilitates the isolation and characterization of emerging viruses that are unable to proliferate in traditional 2D-cultured cells. Notably, the antiviral effect of drugs observed using 3D HAE may reflect the actual activity of those drugs in vivo. Here, we tested the anti-H7N9 activity of type I and type III rhIFNs in different culture systems using H7N9 human clinical isolate A/Anhui/1/2013.

The advantage of immunostaining for virus culture is that the cell tropism of the virus can be detected. Ciliated cells possess both α-2,6- and α-2,3-linked sialic acid receptors, whereas non-ciliated cells present mostly α-2,6-linked sialic acid receptors. We observed that H7N9 can infect both ciliated cells and non-ciliated cells because the H7N9 viral particles bound to both avian-type (α-2,3) and human-type (α-2,6) [25]. Confocal images of H7N9-infected HAE cells clearly displayed a reduction in the amount of cilia and the destruction of tight junctions compared with uninfected controls.

In 1986, rhIFN-α2b was approved by US FDA for the clinical treatment of diseases such as hair cell leukemia, AIDS-related kaposi’s sarcoma, and chronic hepatitis B and C, achieving great social and economic benefits. However, long-term use of rhIFN-α2b may cause side effects such as fatigue, fever, anorexia, and depression, which are associated with the wide distribution of rhIFN-α2b receptors. In contrast, type III IFN receptors have a limited distribution, mainly in the epithelial tissues of the lungs and intestines, so the side effects are relatively minor. Previous studies in our lab have shown that the antiviral activity of rhIFN-λ1 is higher than that of rhIFN-λ2 and rhIFN-λ3. Moreover, type I IFN is effective against the influenza 2009 pandemic H1N1 and highly pathogenic H5N1 strains. For the above reasons, rhIFN-α2b and rhIFN-λ1 were chosen to study the antiviral activity against H7N9 influenza A virus in different cell models.

The viral yield peaked on days 6 and 3 post-infection with 1 TCID_50_ of H7N9 virus in A549 cells and 3D HAE cells, respectively. The viral proliferation profiles in these cells were also different after rhIFNs treatment. In addition, there was a dose-effect relationship between the dose of rhIFN-α2b or rhIFN-λ1 and its activity against H7N9. In all three cell types, the antiviral activity of rhIFN-α2b was slightly better than that of rhIFN-λ1 to varying degrees. Together, these results highlight the inconsistencies between the replication kinetics of H7N9 in 2D-cultured cells and 3D HAE cells as well as the differences in the antiviral activity of rhIFNs between different cells.

A549 cells are adenocarcinomic human alveolar basal epithelial cells, and the 3D HAE cells used in our study was derived from human tracheal tissue. So, the tissue sources of the two cell lines are basically the same. The two chains of type I IFNs receptors, IFNAR1 and IFNAR2, and a component of the type III IFN receptor, IL10R2, was expressed in all cells, while IFNLR1, another component of type III IFN receptors, was only expressed in certain cells. It was reported the expression of IFNLR1 in A549 cells and lung tissue were almost similar using northern blot analysis [26], so the expression levels of type I and type III IFN receptors were also consistent. When binding to receptors located on the cell surface, IFN triggers the downstream JAK–STAT signaling pathway and eventually induces the expression of a series of ISGs. These genes target multiple stages of the viral life cycle and exhibit various antiviral activities due to their diverse structures and functions. MX1 [27, 28] and IFITM3 [29, 30] mainly effect viral invasion, while OAS1 [31] and ISG15 [32, 33] act primarily on the viral replication and transcription stages.

In the present study, the expression levels of MX1, OAS1, IFITM3, and ISG15 in A549 cells, 2D HAE cells, and 3D HAE cells were all clearly increased after incubation with rhIFN-α2b or rhIFN-λ1. In the A549 and 2D HAE cells, the expression levels of these four genes were significantly higher in the rhIFN-λ1 group than in the rhIFN-α2b group. However, it is noteworthy that the corresponding result was exactly the opposite in the 3D HAE cells. This indicates that the induction of ISGs by rhIFN-α2b and rhIFN-λ1 is inconsistent between 2D-cultured cells and 3D HAE cells.

It was reported that the expression of the MX1 can be significantly upregulated in A549 cells when treated with rhIFN-λ1 at 10 ng/ml, but treatment with rhIFN-β at 0.01 pg/ml did not increase the MX1 expression level [34]. In this study, we found that MX1 expression increased markedly following treatment with rhIFN-λ1 or rhIFN-α2b in 2D HAE and A549 cells compared with the results for 3D HAE cells. In addition, knockdown of MX1 expression resulted in a remarkable decrease in the expression of ISGs in A549 cells after infected with influenza A virus, thereby increased virus replication [28]. Although the upregulation of IFN-induced IFITM3 observed in this study was relatively low, the key role of this ISG cannot be ignored. Remarkably, IFITM3 can inhibit H7N9 replication early in viral infection [35]. Additionally, it was reported that monomethylation of IFITM3 at Lys-88 is downregulated after treatment rhIFN-α, thereby enhancing IFITM3 activity and inhibiting influenza virus [36]. Furthermore, the overexpression of IFITM proteins can rigidify the cell membrane, thus markedly inhibiting viral membrane fusion and entry [37]. IFITM3 plays an important role in the pathogenesis of the influenza disease, and it may serve as a potential target for treatment and management of influenza-associated infection.

A recent study by Ilyushina and colleagues [34] showed that a combination of the NA inhibitor oseltamivir carboxylate and rhIFN-λ1 had a strong synergistic interaction. This discovery suggests that rhIFN combined with NA inhibitors might be a more effective therapeutic strategy against H7N9 infection. Our future work will investigate this combination therapy in appropriate cell lines and animal models.

## Conclusions

The H7N9 cell tropism on 3D HAE cells was observed and anti-H7N9 bioactivity of rhIFN-α2b and rhIFN-λ1 on different cell culture systems was tested. Our results indicate that H7N9 can infect both ciliated cells and non-ciliated cells, causing the cilia and tight junction damage. Additionally, the replication kinetics of H7N9 in 2D-cultured cells and 3D HAE cells was different. Both rhIFN-α2b and rhIFN-λ1 have antiviral activity against H7N9 on A549 cells, 2D HAE cells, and 3D HAE cells, and the protective effects were related to the induction of ISGs.

## Abbreviations

3D: Three-dimensional
ALI: Air–liquid interface
CPE: Cytopathic effect
HAE: Human airway epithelium
HE: Hematoxylin-eosin
IFN: Interferon
ISG: Interferon-stimulated gene
NA: Neuraminidase
ZO-1: Zonula occludens-1
